# Multivariate modular metabolic engineering for enhanced l-methionine biosynthesis in *Escherichia coli*

**DOI:** 10.1186/s13068-023-02347-7

**Published:** 2023-06-13

**Authors:** Zhongcai Li, Qian Liu, Jiahui Sun, Jianjian Sun, Mingjie Li, Yun Zhang, Aihua Deng, Shuwen Liu, Tingyi Wen

**Affiliations:** 1 State Key Laboratory of Microbial Resources, Institute of Microbiology, Chinese Academy of Sciences, Beijing, 100101 China; 2 University of Chinese Academy of Sciences, Beijing, 100049 China; 3 National Laboratory of Biomacromolecules, Institute of Biophysics, Chinese Academy of Sciences, Beijing, 100101 China; 4 College of Life Sciences, Hebei University, Baoding, 071002 China; 5 Savaid Medical School, University of Chinese Academy of Sciences, Beijing, 100049 China; 6 China Innovation Academy for Green Manufacture, Chinese Academy of Sciences, Beijing, 100049 China

**Keywords:** l-Methionine, *Escherichia coli*, Metabolic engineering, Multivariate modular, Cystathionine γ-synthase

## Abstract

**Background:**

l-Methionine is the only bulk amino acid that has not been industrially produced by the fermentation method. Due to highly complex and strictly regulated biosynthesis, the development of microbial strains for high-level l-methionine production has remained challenging in recent years.

**Results:**

By strengthening the l-methionine terminal synthetic module via site-directed mutation of l-homoserine O-succinyltransferase (MetA) and overexpression of *metA*^fbr^, *metC*, and *yjeH*, l-methionine production was increased to 1.93 g/L in shake flask fermentation. Deletion of the *pykA* and *pykF* genes further improved l-methionine production to 2.51 g/L in shake flask fermentation. Computer simulation and auxotrophic experiments verified that during the synthesis of l-methionine, equimolar amounts of l-isoleucine were accumulated via the elimination reaction of cystathionine γ-synthetase MetB due to the insufficient supply of l-cysteine. To increase the supply of l-cysteine, the l-cysteine synthetic module was strengthened by overexpression of *cysE*^fbr^, *serA*^fbr^, and *cysDN*, which further increased the production of l-methionine by 52.9% and significantly reduced the accumulation of the byproduct l-isoleucine by 29.1%. After optimizing the addition of ammonium thiosulfate, the final metabolically engineered strain MET17 produced 21.28 g/L l-methionine in 64 h with glucose as the carbon source in a 5 L fermenter, representing the highest l-methionine titer reported to date.

**Conclusions:**

In this study, a high-efficiency strain for l-methionine production was derived from wild-type *Escherichia coli* W3110 by rational metabolic engineering strategies, providing an efficient platform for the industrial production of l-methionine.

**Supplementary Information:**

The online version contains supplementary material available at 10.1186/s13068-023-02347-7.

## Background

l-Methionine is an essential amino acid and is widely used in food, medicine, feed, and other industries [1, 2]. Currently, l-methionine is mainly synthesized by chemical methods, which use volatile and toxic hydrocyanic acid, acrolein, and methanethiol as substrates and cause severe environmental pollution [3]. Therefore, there has been much interest in the production of l-methionine via environmentally friendly and reproducible fermentation by employing metabolically engineered microorganisms.

Several metabolic engineering efforts to efficiently produce l-methionine from glucose in *Escherichia coli* or *Corynebacterium glutamicum* have been reported (Table 1). The major strategies for constructing l-methionine producers include alleviating feedback inhibition, overexpressing key enzymes, blocking competing pathways, increasing the supply of cofactors, and enhancing l-methionine transport. For instance, inactivating the l-methionine transcriptional repressor MetJ and releasing the feedback inhibition of l-homoserine *O*-succinyltransferase MetA result in the initial accumulation of l-methionine [4, 5]. Overexpressing the l-methionine efflux protein YjeH or BrnFE and deleting the l-threonine and l-lysine pathways significantly increased the production of l-methionine [6, 7]. Enhancing the NADPH supply by modifying the pentose phosphate pathway increased l-methionine production [8, 9].

**Table 1:** Overview of metabolic engineering studies on the production of l-methionine by *C. glutamicum* and *E. coli*

Host strain	Genotype	Auxotroph or not	Titer (g/L)	References
*E. coli* W3110	Δ*thrBC*ΔmetJ metK^*^/pMW118-P_*thr*_-metA^*^	Yes	0.24 (Shake flask)	Usuda and Kurahashi 2005 [5]
*C. glutamicum* ATCC13032	Δ*thrB*Δ*mcbR*/pJYW-4-*hom*^fbr^-*lysC*^fbr^-*brnFE*	Yes	6.3 (3 L fed-batch,)	Qin, et al. 2015 [6]
*C. glutamicun* ATCC13032	Δ*metD*Δ*thrB dapA*^A1G^ *lysC*^fbr^ *pyc*^fbr^ *zwf*^fbr^ *gnd*^fbr^	Yes	6.85 (Shake flask)	Li, et al. 2016 [8]
*E. coli* W3110	Δ*thrBC*Δ*metJ*Δ*lysA*/ pTrc99A-metA^*^-*yjeH*	Yes	9.75 (5 L fed-batch)	Huang, et al. 2017 [7]
*C. glutamicun* ATCC13032	*zwf*^fbr^ *gnd*^fbr^/pLY-4-*gapC*	No	0.64 (N)	Liu, et al. 2022 [9]
*E. coli* W3110	Δ*thrBC*Δ*metJ*Δ*lysA* Trc-*metH* Trc-*metF* Trc-*cysE* Trc-*serB* Trc-*serC*/pTrc99A-metA^*^-*yjeH*- *serA*^fbr^-*gltA*	Yes	17.74 (5 L fed-batch)	Niu, et al. 2023 [10]

“*” means mutation of the gene; “Δ” means deletion of genes; “N” means no description

Despite the above efforts to improve l-methionine production by fermentation, the highest titer of l-methionine produced by *C. glutamicum* was 6.85 g/L [9] and *E. coli* was 17.74 g/L [10]. There remain some limitations to these metabolic engineering strategies, for example, the exogenous addition of l-lysine and l-threonine was needed during fermentation due to auxotroph, which increased the cost of l-methionine production and process complexity [6, 7]. Moreover, a recombinant plasmid was used to express key genes in those strains. However, antibiotics and inducers are not conducive to scale-up fermentation [7, 10]. Therefore, developing a non-auxotrophic and plasmid-free strain will be beneficial for the biological industrial production of l-methionine.

In this study, a highly efficient l-methionine producer was derived from *E. coli* W3110 using rational metabolic engineering strategies. The CRISPR/Cas9 system was used to modify the related modules for l-methionine synthesis, and the specific reasons for the accumulation of the byproduct l-isoleucine were analyzed and discussed herein. The enhancement of the l-cysteine synthetic module further increased l-methionine production and significantly reduced l-isoleucine accumulation. The final engineered strain MET17 produced 21.28 g/L l-methionine in a 5 L bioreactor.

## Results and discussion

### Strengthening the l-methionine terminal synthetic module

**Fig. 1:**
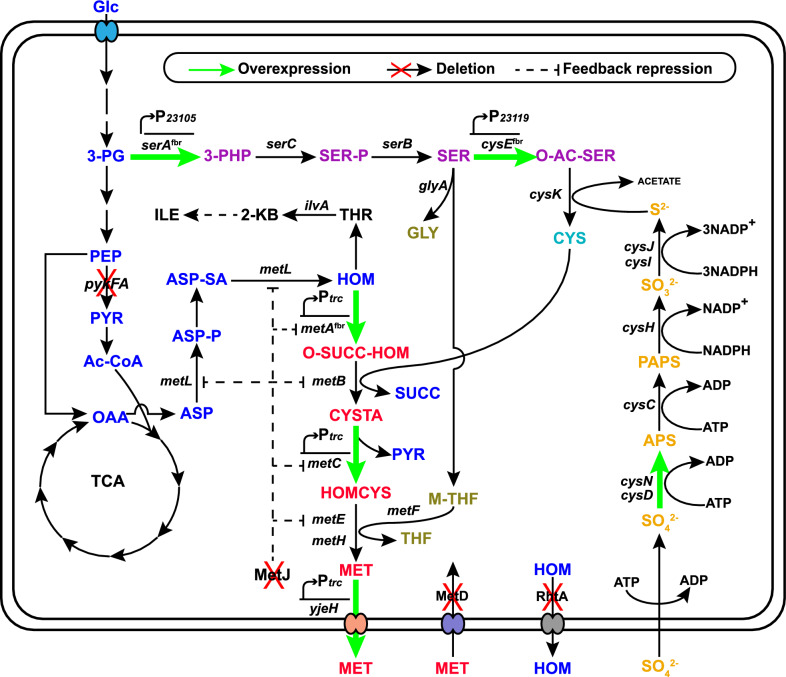
Metabolic strategies for enhancing l-methionine production in *E.coli*. Glc, glucose; 3-PG, glyceraldehyde-3-phosphate; PEP, phosphoenolpyruvate; PYR, pyruvate; Ac-CoA, acetyl coenzyme A; OAA, oxaloacetate; ASP, l-aspartate; ASP-P, aspartyl-β-phosphate; ASP-SA, aspartyl-β-semialdehyde; HOM, l-homoserine; O-SUCC-HOM, *O*-succinyl-l-homoserine; CYSTA, l-cystathionine; HOMCYS, l-homocysteine; MET, l-methionine; 3-PHP, 3-Phosphonooxypyruvate; SER-P, 3-phosphoserine; SER, l-serine; O-AC-SER, *O*-acetyl-l-serine; CYS, l-cysteine; APS, adenosine 5-phosphosulfate; PAPS, 3-phosphoadenosine 5-phosphosulfate; GLY, l-glycine; M-THF, 5-methyltetrahydrofolate; THF, tetrahydrofolate; THR, l-threonine; 2-KB, 2-ketobutyrate; ILE, l-isoleucine; *pykFA*, pyruvate kinase; *metL*, fused aspartate kinase/homoserine dehydrogenase 2; *metA*, l-homoserine *O*-succinyltransferase; *metB*, cystathionine γ-synthase; *metC*, cystathionine β-lyase; *metE*/*H*, homocysteine methyltransferase; *yjeH*, l-methionine exporter; MetD, l-methionine absorption transporter operon; RhtA, l-homoserine exporter; *serA*, phosphoglycerate dehydrogenase; *serC*, phosphoserine aminotransferase; *serB*, phosphoserine phosphatase; *cysE*, serine *O*-acetyltransferase; *cysK*, cysteine synthase; *cysN*, sulfate adenylyltransferase subunit 1; *cysD*, sulfate adenylyltransferase subunit 2; *cysC*, adenylylsulfate kinase; *cysH*, phosphoadenosine phosphosulfate reductase; *cysJ*, sulfite reductase (NADPH) flavoprotein alpha-component; *cysI*, sulfite reductase (NADPH) flavoprotein beta-component

**Fig. 2:**
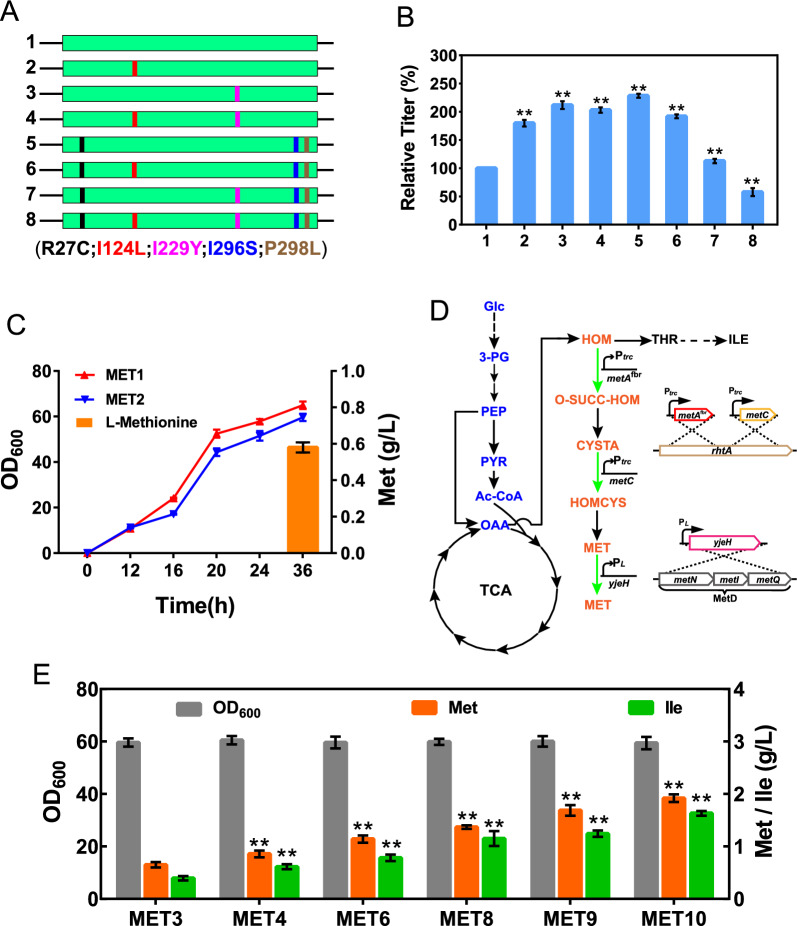
Metabolic engineering modification of the l-methionine terminal synthetic module. **A** Schematic diagram of MetA mutation sites. **B** The relative l-methionine titer of different mutant MetA-overexpressing strains in shake flask fermentation. **C** Production of l-methionine and growth of the MET1 and MET2 strains. **D** Flow chart of chromosomal integration for the *metA*^fbr^, *metC*, and *yjeH* genes. **E** l-Methionine and l-isoleucine production and OD_600_ of MET3, MET4, MET6, MET8, MET9, and MET10. Data are presented as the mean values with the standard deviation from three biological replicates. One-way analysis of variance (ANOVA) was used to determine significant differences (* *p* < 0.05, ** *p* < 0.01)

In *E. coli*, the biosynthesis pathway from l-homoserine to l-methionine forms the l-methionine terminal synthetic module, where the components required (including the carbon skeleton, sulfur, and methyl group) are assembled into l-methionine and transported out of the cell (Fig. 1). Removing the feedback inhibition of key enzymes in metabolic pathways is usually the first step in the construction of genetically engineered strains [11]. As the first key enzyme in the l-methionine terminal synthetic module, l-homoserine *O*-succinyltransferase (MetA, encoded by *metA*) is feedback inhibited by l-methionine and *S*-adenosylmethionine (SAM) [12] and is extremely sensitive to many stress conditions (e.g., thermal, oxidative, or acidic stress) [13–15]. To optimize MetA performance, we combined the previously reported thermostable mutations I124L and I229Y [16] with the feedback-resistant (fbr) mutations I296S-P298L-R27C [5] by site-directed mutagenesis (Fig. 2A). Seven MetA mutants were constructed by plasmid pBR322 and overexpressed in MET1 (in which a transcriptional repressor of the l-methionine biosynthesis genes, encoded by *metJ*, was deleted from the wild-type *E. coli* W3110). The expression of different MetA mutants was detected by SDS-PAGE (Additional file 1: Figure S1). Shake flask fermentation showed that except for MetA^I124L, I229L, R27C-I296S-P298L^, all the MetA mutants exhibited significantly increased l-methionine production, and the MetA ^R27C-I296S-P298L^ (*metA*^fbr^) mutant exhibited the highest relative titer of l-methionine, which was 2.3-fold higher than that of the wild type (Fig. 2B). These results demonstrated that combining all of these mutations was not suitable for l-methionine production and significantly inhibited l-methionine synthesis. Then, we introduced the R27C, I296S, and P298L mutations into the chromosome of MET1 to obtain the MET2 strain (MET1*metA*^fbr^). As shown in Fig. 2C, MET2 produced 0.58 g/L l-methionine at 36 h, and its growth was slightly reduced compared with that of MET1, while MET1 did not exhibit l-methionine accumulation. When we drafted this manuscript, Hye-Young Sagong et al. [17] reported the crystal structure of MetA from *E. coli* and identified a putative l-methionine inhibitor binding site T242 located in the vicinity of the substrate binding site. The T242A variant retained more than 70% of its activity with the addition of 5 mM l-methionine and the *Ki* was 17.4 mM, which significantly reduced the feedback inhibition compared with the wild type (*Ki* was 2.44 mM). This provides valuable knowledge for the improvement of l-methionine production. Especially, the combination of T242A with I296S-P298L-R27C could be useful for further study of the effect on l-methionine synthesis.

To further improve l-methionine production, a copy of *metA*^fbr^ driven by the strong P_*trc*_ promoter was integrated into the *rhtA* site (encoding an effective exporter of l-homoserine, a precursor for l-methionine synthesis) of MET2 to obtain the MET4 strain (Fig. 2D). The *rhtA* gene was deleted from strain MET2 to generate MET3 as a control. Shake flask fermentation results showed that MET3 and MET4 produced 0.63 g/L and 0.86 g/L l-methionine, which were 8.6% and 48.3% higher than the amount produced by MET2, respectively (Fig. 2E). While MET3 and MET4 also accumulated 0.39 g/L and 0.61 g/L l-isoleucine, respectively. To investigate other key genes that affect l-methionine synthesis in the l-methionine terminal synthetic module, the *metBL*, *metC*, and *metF* genes of MET4 were individually upregulated by replacing the native promoter with the strong promoter P_*trc*_ to obtain the MET5, MET6, and MET7 strains. As shown in Additional file 1: Figure S2, the relative l-methionine titer of the MET6 strain (with upregulation of *metC*) was 29.1% higher than that of the MET4 strain. Upregulation of *metBL* (MET5) and *metF* (MET7) expression significantly reduced the relative l-methionine titer by 84.9% and 22.1%, respectively, which might be due to the upregulation of these genes affecting the balance of intracellular metabolism, which is consistent with the results of previous studies [18]. Then, we integrated a copy of *metC* driven by the strong promoter P_*trc*_ into the *rhtA* site of MET6 to obtain strain MET8. Shake flask fermentation results showed that MET8 produced 1.36 g/L l-methionine, which was 21.1% higher than the amount produced by MET6 (Fig. 2E). Notably, MET8 also accumulated 1.14 g/L l-isoleucine. The l-isoleucine pathway is a competing pathway for l-methionine synthesis in *E. coli*, and the overexpression of *metA*^fbr^ and *metC* in the MET8 strain would enlarge the carbon flux from l-homoserine to l-methionine and reduce the metabolic flux to l-isoleucine pathway (Fig. 2D). However, the accumulation of l-isoleucine was also increased, so we speculated that there might be other potential pathways for l-isoleucine synthesis.

Finally, l-methionine efflux was enhanced by blocking the absorption transporter MetD (encoded by the *metN*, *metI*, and *metQ* genes) [19] and overexpressing the efflux protein YjeH (encoded by *yjeH*) [20] in MET8 strain. The MET9 strain (MET8Δ*metD*) and MET10 strain (MET8Δ*metD*::P_*L*_-*yjeH*) were constructed, respectively (Fig. 2D). Shake flask fermentation results showed that MET9 and MET10 produced 1.68 g/L and 1.93 g/L l-methionine, which were 23.5% and 41.9% higher, respectively, than the amount produced by the MET8 strain. As YjeH can also transport branched-chain amino acids [20], the accumulation of the byproduct l-isoleucine also increased to 1.24 g/L and 1.63 g/L in MET9 and MET10, respectively (Fig. 2E).

### Coupling of the central carbon metabolism module with the l-methionine terminal synthetic module

**Fig. 3:**
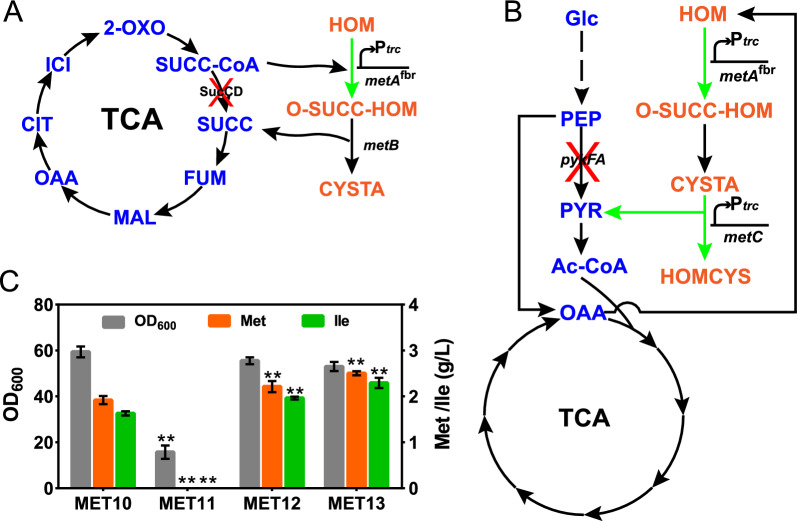
Effect of metabolic coupling strategy on l-methionine production. **A** Coupling of the central carbon metabolism module to the l-methionine terminal synthetic module by deleting the succinyl-CoA synthetase *sucCD.* **B** Coupling of the central carbon metabolism module with the l-methionine terminal synthetic module by deleting the *pykF* and *pykA* genes. **C** l-Methionine and l-isoleucine production and OD_600_ of MET10, MET11, MET12, and MET13. Data are presented as the mean values with the standard deviation from three biological replicates. One-way analysis of variance (ANOVA) was used to determine significant differences (* *p* < 0.05, ** *p* < 0.01)

The l-methionine synthesis pathway in *E. coli* is complex and is regulated by multiple factors [18]. Some metabolic pathways share the same substrate and product. For instance, succinyl-CoA and l-homoserine are converted to *O*-succinyl-l-homoserine, and succinic acid is released during the process of *O*-succinyl-l-homoserine being converted to cystathionine, catalyzed by cystathionine γ-synthase (encoded by *metB*), which is consistent with the conversion of succinyl-CoA to succinic acid (catalyzed by succinyl-CoA synthetase, encoded by *sucCD*) in the TCA cycle of the central carbon metabolism module (Fig. 3A). On the other hand, the decomposition of cystathionine to homocysteine by cystathionine β-lyase (encoded by *metC*) releases pyruvate, the same reaction product formed by pyruvate kinase (encoded by *pykF* and *pykA*) in glycolysis in the central carbon metabolism module (Fig. 3B). Kind et al. [21] and Ruan et al. [22] used a metabolic coupling strategy to delete the *sucCD* genes, which coupled the TCA cycle with the l-lysine and SAM synthesis pathways in *C. glutamicum* and *Bacillus amyloliquefaciens*, respectively, and significantly increased the production of these compounds. To verify the effect of these couplings on l-methionine production, the *sucCD*, *pykA*, and *pykF* genes of MET10 were deleted to obtain the MET11 (*sucCD*), MET12 (*pykA*), and MET13 (*pykApykF*) strains, respectively. As shown in Fig. 3C, the growth of the MET11 strain was significantly inhibited, and l-methionine accumulation was not detected. This might be attributed to the l-methionine terminal synthetic module not compensating for the TCA cycle to maintain normal cell growth in the *sucCD*-deleted strain, which was also demonstrated in *B. amyloliquefaciens* [22]. In contrast, MET12 and MET13 produced 2.38 g/L and 2.51 g/L l-methionine, respectively, which were 23.3% and 30.1% higher than the amount produced by MET10. In addition, 1.97 g/L and 2.27 g/L l-isoleucine as a byproduct also accumulated in the MET12 and MET13 strains. Deletion of the *pykA* and *pykF* genes increased l-methionine precursor synthesis and coupled l-methionine synthesis with cell growth. Due to the enhancement of the l-methionine terminal synthetic module and pyruvate release being commonplace in synthetic pathways [23, 24], cell growth was not affected.

### In silico simulation of flux distribution for l-isoleucine accumulation

**Fig. 4:**
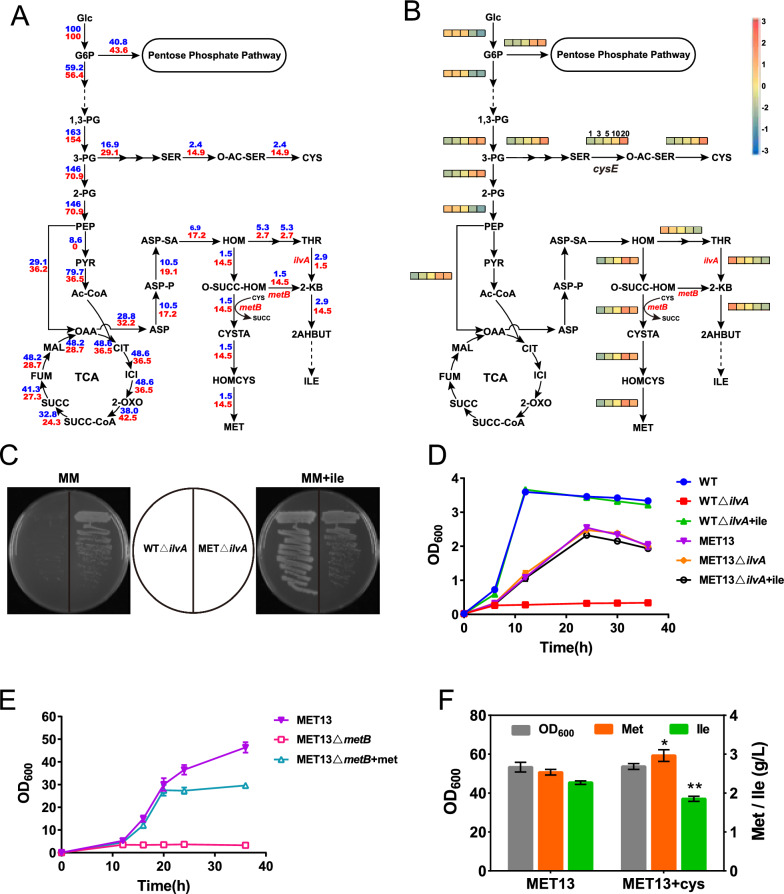
In silico simulation of the metabolic flux distribution in the wild-type strain and l-methionine overproducer by flux balance analysis. **A** The metabolic flux distribution in the WT strain (blue) and l-methionine overproducer (red). **B** The impacts of increased CysE reaction flux on intracellular metabolic flux by FBA using l-methionine synthase as the objective function with 20% theoretical maximum biomass constraint. The CysE reaction flux was set to 1.0-, 3.0-, 5.0-, 10.0-, and 20.0-fold (from left to right) compared to that in the WT strain. **C** Strains WTΔ*ilvA* and MET13Δ*ilvA* on solid M9 medium with or without 0.5 g/L l-isoleucine supply. **D** Growth of WT, WTΔ*ilvA*, MET13, and MET13Δ*ilvA* in M9 medium with or without 0.5 g/L l-isoleucine supply. **E** Growth of MET13 and MET13Δ*metB* in fermentation medium with or without 0.5 g/L l-methionine supply. **F** l-methionine and l-isoleucine production and OD_600_ of MET13 and MET13 with l-cysteine supply. Data are presented as the mean values with the standard deviation from three biological replicates. One-way analysis of variance (ANOVA) was used to determine significant differences (* *p* < 0.05, ** *p* < 0.01)

Although the production of l-methionine was significantly promoted by modifying the l-methionine terminal synthetic module, considerable l-isoleucine (2.27 g/L, Fig. 3C) was accumulated by MET13, indicating that we should shift the metabolic flux from l-isoleucine to the synthesis of l-methionine. In wild-type *E. coli*, the metabolic flux in the l-homoserine node was partitioned partially to *O*-succinyl-l-homoserine for l-methionine synthesis and partially to l-threonine for l-isoleucine formation (Fig. 1). Since a common strategy to block the l-isoleucine pathway is to delete *ilvA*, we deleted the *ilvA* gene in the MET13 strain to obtain the MET13Δ*ilvA* strain, and deletion of *ilvA* in the wild-type *E. coli* W3110 (WTΔ*ilvA*) was performed as a control. Surprisingly, WTΔ*ilvA* was found to be l-isoleucine auxotrophic in M9 minimum medium without l-isoleucine, but MET13Δ*ilvA* did not exhibit this trait (Fig. 4C, D). These results indicated that the synthesis of l-isoleucine occurs via other pathways.

To investigate the underlying reason, the relationships between l-methionine flux and biomass as well as l-isoleucine accumulation were investigated by flux balance analysis (FBA) [25] based on the *i*Y75_1357 model of *E. coli* W3110 [26]. The intracellular metabolic fluxes in the WT strain were calculated using biomass as the objective function. For the l-methionine producer, the lowest biomass was restricted to 20% of the theoretical maximum, and the metabolic fluxes were calculated using l-methionine synthase as the objective function. The comparison of metabolic flux distribution between the WT strain and l-methionine producer showed the most important difference in the flux redistribution as being at the l-cysteine synthetic module and l-methionine terminal synthetic module. The flux from *O*-acetyl-l-serine entering into l-cysteine increased 6.2-fold in the l-methionine producer compared to the WT strain. In contrast, the flux from l-homoserine to l-threonine in the l-methionine producer dropped nearly twofold, and there was a 9.7-fold increase in the flux redirected toward the l-methionine terminal synthetic module (Fig. 4A). As previously reported, when the l-cysteine supply is insufficient, cystathionine γ-synthase (encoded by *metB*) catalyzes the conversion of *O*-succinyl-l-homoserine (OSHS) to 2-ketobutyrate (2-KB) via an elimination reaction and then forms l-isoleucine [27, 28]. The flux from OSHS to 2-KB also increased 9.7-fold in the l-methionine producer (Fig. 4A). Considering that the *cysE*-catalyzed reaction is a major control switch in the l-cysteine synthesis pathway of *E. coli* [29, 30], the effects of the *cysE*-catalyzed reaction flux change on the carbon flux distribution and l-methionine synthesis were investigated using FBA under 20% of the theoretical maximum biomass constraint. When the *cysE*-catalyzed reaction flux improved tenfold, the l-methionine productivity reached a relative maximum (Fig. 4B). Meanwhile, the flux toward 2-KB decreased, which indicated the diversion of more OSHS toward l-methionine synthesis.

To verify the simulation, we performed fermentation experiments with MET13 lacking the *metB* (MET13Δ*metB*) gene and with the exogenous addition of l-cysteine. As shown in Fig. 4E, when the *metB* gene of MET13 was deleted, the cells showed l-methionine auxotroph and l-methionine supplementation restored the growth of MET13Δ*metB*. l-Isoleucine was also not detected in MET13Δ*metB* after fermentation (data not shown). After l-cysteine was added during the fermentation process of MET13, the accumulation of l-isoleucine decreased by 18.5%, and the production of l-methionine increased by 16.5% (Fig. 4F). These results indicated that the l-cysteine supply was insufficient during l-methionine fermentation and that the accumulation of l-isoleucine occurred mainly due to the elimination reaction of MetB in the l-methionine terminal synthetic module rather than the l-threonine synthetic pathway.

### Strengthening the l-cysteine synthetic module

**Fig. 5:**
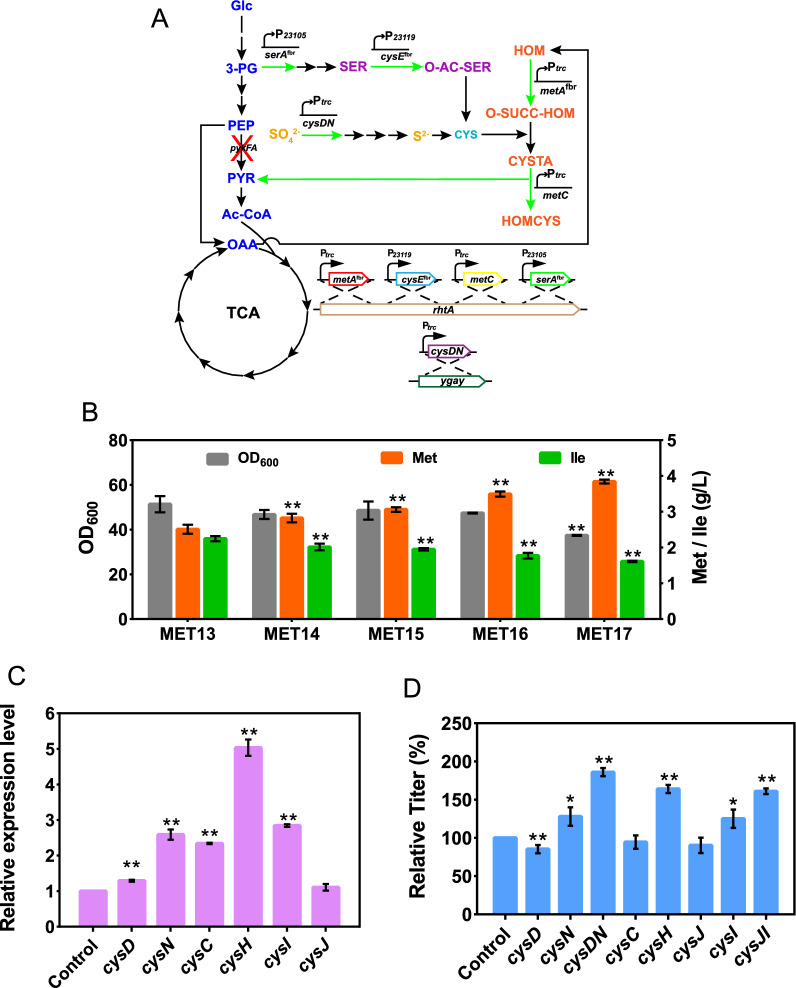
Metabolic engineering modification of the l-cysteine synthetic module. **A** A schematic representation of the enhancement of the l-cysteine synthetic module. **B** l-methionine and l-isoleucine production and OD_600_ of MET13, MET14, MET15, MET16, and MET17. **C** Relative transcript levels of related genes of MET15 in shake flask cultivation. **D** Effect of overexpression of key genes in the sulfur assimilation pathway on l-methionine production. Data are presented as the mean values with the standard deviation from three biological replicates. One-way analysis of variance (ANOVA) was used to determine significant differences (* *p* < 0.05, ** *p* < 0.01)

In *E. coli*, the l-cysteine synthetic module involves both carbon metabolism and sulfur metabolism [31]. In terms of carbon metabolism, glyceraldehyde 3-phosphate (3-PG) is catalytically acted upon by phosphoglycerate dehydrogenase (PGDH, encoded by *serA*), phosphoserine aminotransferase (encoded by *serC*), phosphoserine phosphatase (encoded by *serB*), and l-serine acetyltransferase (SAT, encoded by *cysE*) to form *O*-acetyl-l-serine (OAS) (Fig. 1). SAT and PGDH are the two rate-limiting enzymes of l-cysteine synthesis, which are inhibited by l-cysteine and l-serine, respectively [30, 32]. To increase the l-cysteine supply, we first integrated a copy of feedback-resistant *cysE*^fbr^ (cysE^M201R^) [33, 34] which was controlled by the P_*23119*_ promoter into the chromosome of MET13, with wild-type *cysE* as a control, to obtain the MET14 (P_*trc*_-*cysE*) and MET15 (P_*trc*_-*cysE*^fbr^) strains, respectively (Fig. 5A). As shown in Fig. 5B, MET14 and MET15 produced 2.82 g/L and 3.06 g/L l-methionine, increasing by 18.5% and 28.6% than MET13, respectively. While the accumulation of l-isoleucine was 2.02 g/L and 1.95 g/L, decreasing by 21.1% and 23.8% than MET13, respectively. Then, we integrated a copy of feedback-resistant *serA*^fbr^ (*serA*^H344A−N346A^) [35] into the chromosome of MET15. Although we initially attempted to integrate *serA*^fbr^ with the strong promoter P_*23119*_ control, the experiment consistently failed. The failure might have been due to the excessive activity of SAT and PGDH resulting in the overconsumption of carbon sources and destruction of the balance of the glycolysis and l-cysteine synthetic module. Therefore, we used a relatively weak promoter, P_*23105*_ (Fig. 5A). The resultant MET16 strain produced 3.49 g/L l-methionine and 1.78 g/L l-isoleucine, representing a 14.1% increase in l-methionine production compared to that in MET15 and a 12.4% decrease in l-isoleucine production, with a slight decrease in biomass (Fig. 5B). Since the expression of sulfur assimilation-related genes in *E. coli* is activated by OAS [29], we confirmed the expression of these genes in MET16 by RT‒PCR, and the results showed that the expression of sulfur reduction genes was significantly upregulated (Fig. 5C).

**Fig. 6:**
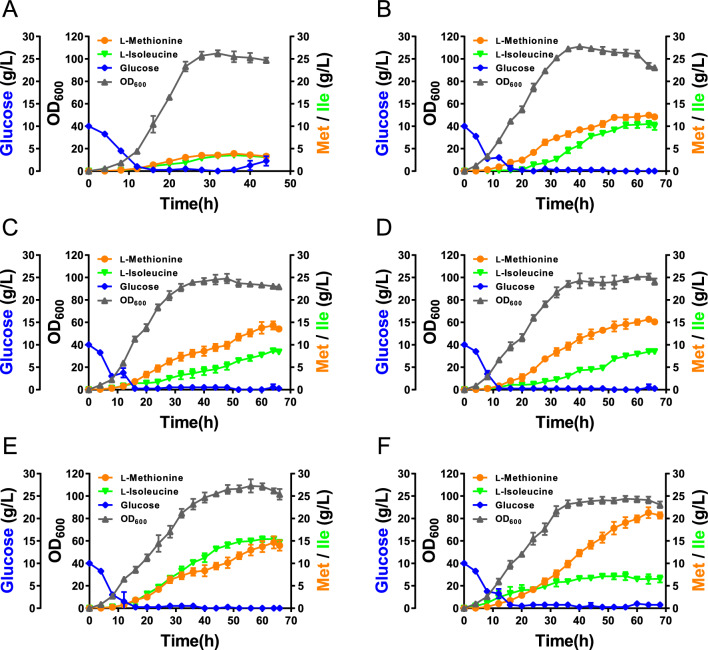
Fed-batch fermentation process curve in a 5 L bioreactor. **A** Without sulfate or thiosulfate feeding during fed-batch cultivation of the MET17 strain. **B** With sodium thiosulfate feeding during fed-batch cultivation of the MET17 strain. **C** With ammonium thiosulfate feeding during fed-batch cultivation of the MET17 strain. **D** With ammonium sulfate and sodium thiosulfate feeding during fed-batch cultivation of the MET17 strain. **E** With ammonium sulfate and ammonium thiosulfate feeding during fed-batch cultivation of the MET13 strain. **F** With ammonium sulfate and ammonium thiosulfate feeding during fed-batch cultivation of the MET17 strain

In terms of sulfur metabolism, sulfate is often used as a sulfur source for *E. coli* [36]. It can only be assimilated by the sulfate assimilation pathway which includes sulfate adenylyltransferase (encoded by *cysD* and *cysN*), adenylyl-sulfate kinase (encoded by *cysC*), phosphoadenosine phosphosulfate reductase (encoded by *cysH*) and sulfite reductase (encoded by *cysJ* and *cysI*) [37] (Fig. 5). To investigate the effect of key enzymes in the sulfate assimilation pathway on l-methionine production, we overexpressed the key genes *cysD*, *cysN*, *cysH*, *cysC*, *cysJ*, and *cysI* from the pACYC184 plasmid (Fig. 5A). Simultaneously, we also designed constructs for the coexpression of *cysDN* and *cysJI,* the two enzyme subunits. Then, the eight constructed plasmids were transferred into MET16, with the pACYC184 plasmid used as the control. The effects of these genes on l-methionine production were compared by shake flask fermentation. As shown in Fig. 5D, *CysN*, *CysDN*, *CysH, CysI*, and *CysJI* significantly increased the accumulation level of l-methionine. The overexpression of *cysDN* produced the highest relative titer of l-methionine, a 1.86-fold improvement in comparison with the control strain. These results indicate that *cysDN* plays an important role in the sulfur assimilation pathway. Then, we integrated a copy of *cysDN* under the control of the P_*trc*_ promoter into the chromosome of MET16 to obtain strain MET17. The results of shake flask fermentation showed that MET17 produced 3.84 g/L l-methionine and 1.61 g/L l-isoleucine, representing a 10.0% increase in l-methionine production compared to that in MET16 and a 9.0% decrease in l-isoleucine production, with a significant decrease in biomass (Fig. 6C). The decrease in biomass might have been due to the increased consumption of ATP and NADPH by the enhanced sulfur assimilation pathway [38]. Enhancement of the l-cysteine ​​synthetic module increased l-methionine production by 52.9% and decreased l-isoleucine accumulation by 29.1% compared with that in MET13, indicating that sulfur metabolism plays a crucial role in l-methionine synthesis.

### Fed-batch fermentation for l-methionine in a 5-L fermenter

Different sulfur sources greatly affect the theoretical yield of l-methionine because sulfur assimilation requires a large amount of ATP and NADPH [39, 40]. As previously reported, thiosulfate is a much more effective sulfur source for l-methionine fermentation [6, 38]. To obtain a better sulfur source, we compared the effects of sodium thiosulfate and ammonium thiosulfate on l-methionine production in the MET17 strain in a 5 L bioreactor (Fig. 6). Without sulfur source feeding, MET17 only produced 3.90 g/L l-methionine and 3.53 g/L l-isoleucine at 36 h, with a maximum biomass of 104.9 (OD_600_) (Fig. 6A). In contrast, with thiosulfate feeding, the fermentation period was extended from 36 to 64 h and the l-methionine production was significantly increased. With sodium thiosulfate feeding, MET17 produced 12.45 g/L l-methionine and 10.49 g/L l-isoleucine, with a maximum biomass of 111 (Fig. 6B). With ammonium thiosulfate feeding, MET17 produced 14.32 g/L l-methionine and 8.64 g/L l-isoleucine, with a maximum biomass of 99.1 (Fig. 6C). These results indicated that feeding with sulfur sources is essential during l-methionine fermentation and that ammonium thiosulfate is better than sodium thiosulfate for l-methionine production. However, excessive thiosulfate is toxic to cells [41]. As the feeding concentration of ammonium thiosulfate increased, cell growth and l-methionine production were severely inhibited (Additional file 1: Figure S3). Therefore, we subsequently chose the combination of ammonium sulfate and thiosulfate. With ammonium sulfate and sodium thiosulfate feeding, MET17 produced 15.68 g/L l-methionine and 8.46 g/L l-isoleucine, with a maximum biomass of 100.7 (Fig. 6D). With ammonium sulfate and ammonium thiosulfate feeding, MET17 produced 21.28 g/L l-methionine and 7.16 g/L l-isoleucine, with a maximum biomass of 97.5 (Fig. 6F), which represented the highest l-methionine titer ever reported and the yield and productivity were 0.12 mol/mol glucose and 0.33 g/L/h, respectively. Under the same conditions, the maximum biomass of MET17 was 10.6% lower than that of MET13 (109.1), and the maximal l-methionine titer of MET17 was 45.3% higher than that of MET13 (14.65 g/L). The maximal l-isoleucine titer of MET17 was 53.6% lower than that of MET13 (15.42 g/L) (Fig. 6E). These results further indicated that the enhancement of the l-cysteine synthetic module had an important effect on l-methionine biosynthesis. Recently, Niu et al. reported a non-auxotrophic l-methionine-producing strain by dynamically regulating the l-lysine biosynthesis pathway, enhancing the central metabolic pathway and l-cysteine catabolic pathway, and finally producing 17.74 g/L l-methionine [10]. However, their l-methionine-producing strain used recombinant plasmid for overexpressing *metA*^fbr^, *yjeH*, *serA*^fbr^, *gltA,* and *malY* genes. Recombinant plasmid maintenance is considered to be a metabolic burden [42] and is also not conducive to stability in scale-up fermentation [43]. The characteristics of being plasmid-free, inducer-free, and antibiotic-free made MET17 strain more advantageous in fermentation stability, process simplicity, and economic feasibility. Taken together, developing a non-auxotrophic and plasmid-free strain will be beneficial for the biological industrial production of l-methionine.

## Conclusions

**Fig. 7:**
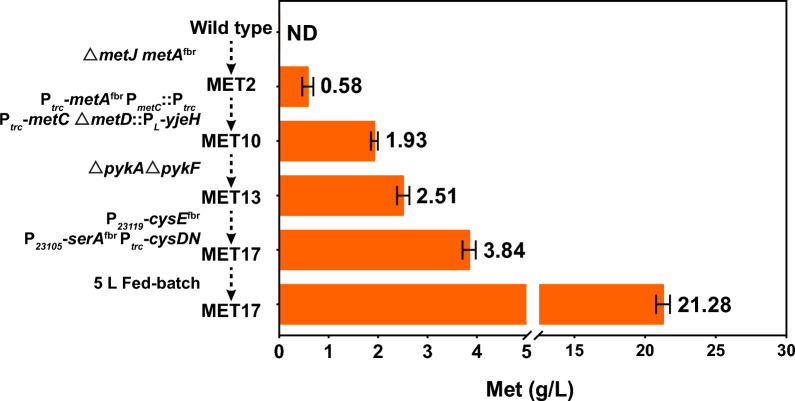
Overview of the strain engineering process. The l-methionine titer was significantly improved by metabolic engineering, from 0.58 g/L in the MET2 strain to 21.28 g/L in the final strain MET17. ND, not detected

In this study, a highly efficient *E. coli* chassis for producing l-methionine from glucose was constructed by strengthening the l-methionine terminal synthetic module, coupling the l-methionine terminal synthetic module with central carbon metabolism module, and strengthening the l-cysteine synthetic module (Fig. 7). Additionally, the specific cause of l-isoleucine accumulation during l-methionine synthesis was elucidated by computer simulation and experiments. The best-performing strain, MET17, produced 3.84 g/L l-methionine in a shake flask and 21.28 g/L in a 5 L fed-batch fermenter, representing the highest l-methionine titer ever reported using rationally engineered microbial cell factories.

## Materials and methods

### Bacterial strains, plasmids, and reagents

All bacterial strains and plasmids used in this study and their relevant characteristics are listed in Table 2 and Additional file 1: Table S1. *E. coli* EC135 was used as a cloning host for plasmid construction, and *E. coli* W3110 was used for metabolic engineering. Q5 Hot Start high-fidelity DNA polymerase (New England BioLabs) was used to amplify donor DNA fragments. The DNA Purification Kit, Bacteria DNA Kit, and Mini Plasmid Kit from TIANGEN BIOTECH were used for DNA preparation. A Q5 Site-Directed Mutagenesis Kit from New England BioLabs was used to construct all pTargetF plasmids. In addition, 2 × M5 Super FastTaq PCR Master Mix from Mei5Bio (Beijing) was used for colony PCR. All primers were synthesized by TIANYI HUI YUAN (Beijing), and the primer sequences are listed in Additional file 1: Table S2. Tryptone and yeast extract were supplied by OXOID. Amino acids and antibiotics were acquired from Sigma-Aldrich. All other chemicals were purchased from Beijing Chemical Works unless otherwise stated.

**Table 2:** Strains used in this study with relevant characteristics

Strains	Relevant characteristics	Sources
EC135	TOP10 Δ*dcm*::*FRT recA*^+^∆*dam*::*FRT,* genotype: *F*^*−*^* mcrA* Δ*(mrr-hsdRMS-mcrBC) φ80 lacZ* Δ*M15* Δ*lacX74 nupG recA1 araD139* Δ*(ara-leu)7697 galE15 galK16 rpsL(Str*^*R*^*) endA1 λ*^*−*^ Δ*dcm*::*FRT recA*^+^Δ*dam*::*FRT*	[44]
W3110	*F-λ-rph-1 INV*(*rrnD,rrnE*)	[45]
MET1	W3110Δ*metJ*	This study
MET2	W3110Δ*metJ metA*^fbr^	This study
MET3	W3110Δ*metJ metA*^fbr^Δ*rhtA*	This study
MET4	W3110Δ*metJ metA*^fbr^Δ*rhtA*::P_*trc*_-*metA*^fbr^	This study
MET5	W3110Δ*metJ metA*^fbr^Δ*rhtA*::P_*trc*_-*metA*^fbr^ P_*metBL*_::P_*trc*_	This study
MET6	W3110Δ*metJ metA*^fbr^Δ*rhtA*::P_*trc*_-*metA*^fbr^ P_*metC*_*::*P_*trc*_	This study
MET7	W3110Δ*metJ metA*^fbr^Δ*rhtA*::P_*trc*_-*metA*^fbr^ P_*metF*_::P_*trc*_	This study
MET8	W3110Δ*metJ metA*^fbr^Δ*rhtA*::P_*trc*_-*metA*^fbr^ P_*metC*_*::*P_*trc*_Δ*rhtA*::P_*trc*_-*metC*	This study
MET9	W3110Δ*metJ metA*^fbr^Δ*rhtA*::P_*trc*_-*metA*^fbr^ P_*metC*_*::*P_*trc*_Δ*rhtA*::P_*trc*_-*metC*Δ*metD*	This study
MET10	W3110Δ*metJ metA*^fbr^Δ*rhtA*::P_*trc*_-*metA*^fbr^ P_*metC*_*::*P_*trc*_Δ*rhtA*::P_*trc*_-*metC* Δ*metD*::P_*L*_-*yjeH*	This study
MET11	W3110Δ*metJ metA*^fbr^Δ*rhtA*::P_*trc*_-*metA*^fbr^ P_*metC*_*::*P_*trc*_Δ*rhtA*::P_*trc*_-*metC* Δ*metD*::P_*L*_-*yjeH*Δ*sucCD*	This study
MET12	W3110Δ*metJ metA*^fbr^Δ*rhtA*::P_*trc*_-*metA*^fbr^ P_*metC*_*::*P_*trc*_Δ*rhtA*::P_*trc*_-*metC* Δ*metD*::P_*L*_-*yjeH*Δ*pykA*	This study
MET13	W3110Δ*metJ metA*^fbr^Δ*rhtA*::P_*trc*_-*metA*^fbr^ P_*metC*_*::*P_*trc*_Δ*rhtA*::P_*trc*_-*metC* Δ*metD*::P_*L*_-*yjeH*Δ*pykA*Δ*pykF*	This study
MET14	W3110Δ*metJ metA*^fbr^Δ*rhtA*::P_*trc*_-*metA*^fbr^ P_*metC*_*::*P_*trc*_Δ*rhtA*::P_*trc*_-*metC* Δ*metD*::P_*L*_-*yjeH*Δ*pykA*Δ*pykF*Δ*rhtA*::P_*23119*_-*cysE*	This study
MET15	W3110Δ*metJ metA*^fbr^Δ*rhtA*::P_*trc*_-*metA*^fbr^ P_*metC*_*::*P_*trc*_Δ*rhtA*::P_*trc*_-*metC* Δ*metD*::P_*L*_-*yjeH*Δ*pykA*Δ*pykF*Δ*rhtA*::P_*23119*_-*cysE*^fbr^	This study
MET16	W3110Δ*metJ metA*^fbr^Δ*rhtA*::P_*trc*_-*metA*^fbr^ P_*metC*_*::*P_*trc*_Δ*rhtA*::P_*trc*_-*metC* Δ*metD*::P_*L*_-*yjeH*Δ*pykA*Δ*pykF*Δ*rhtA*::P_*23119*_-*cysE*^fbr^Δ*rhtA*::P_*23105*_-*serA*^fbr^	This study
MET17	W3110Δ*metJ metA*^fbr^Δ*rhtA*::P_*trc*_-*metA*^fbr^ P_*metC*_*::*P_*trc*_Δ*rhtA*::P_*trc*_-*metC* Δ*metD*::P_*L*_-*yjeH*Δ*pykA*Δ*pykF*Δ*rhtA*::P_*23119*_-*cysE*^fbr^Δ*rhtA*::P_*23105*_-*serA*^fbr^Δ*ygaY*::P_*trc*_-*cysDN*	This study
*E. coli* W3110/p1	*E. coli* W3110 carrying pBR322-*metA*	This study
W3110/p2	*E. coli* W3110 carrying pBR322-*metA*^I124L^	This study
*E. coli* W3110/p3	*E. coli* W3110 carrying pBR322-*metA*^I229Y^	This study
*E. coli* W3110/p4	*E. coli* W3110 carrying pBR322-*metA*^I124L, I229Y^	This study
*E. coli* W3110/p5	*E. coli* W3110 carrying pBR322-*metA*^R27C, I296S, P298L^	This study
*E. coli* W3110/p6	*E. coli* W3110 carrying pBR322-*metA*^R27C, I124L, I296S, P298L^	This study
*E. coli* W3110/p7	*E. coli* W3110 carrying pBR322-*metA*^R27C, I229Y, I296S, P298L^	This study
*E. coli* W3110/p8	*E. coli* W3110 carrying pBR322-*metA*^R27C, I124L, I229Y, I296S, P298L^	This study
*E. coli* W3110/ pBR322	*E. coli* W3110 carrying pBR322	This study
MET16/p9	MET16 carrying pACYC184-*cysD*	This study
MET16/p10	MET16 carrying pACYC184-*cysN*	This study
MET16/p11	MET16 carrying pACYC184-*cysDN*	This study
MET16/p12	MET16 carrying pACYC184-*cysC*	This study
MET16/p13	MET16 carrying pACYC184-*cysH*	This study
MET16/p14	MET16 carrying pACYC184-*cysJ*	This study
MET16/p15	MET16 carrying pACYC184-*cysI*	This study
MET16/p16	MET16 carrying pACYC184-*cysJI*	This study
MET16/ pACYC184	MET16 carrying pACYC184	This study

### Medium and culture conditions

During strain construction, cultures were grown aerobically at 30 °C or 37 °C in lysogeny broth (LB) medium (5 g/L yeast extract, 10 g/L tryptone, 10 g/L NaCl) [46]. To prepare a solid medium, 20 g/L agar powder was added to the LB medium. If necessary, antibiotics were added at the following concentrations: 50 µg/mL kanamycin or 50 µg/mL spectinomycin. L-(+)-arabinose (10 mM final concentration) was used to induce the CRISPR/Cas9 system and Red homologous recombination system during gene deletion and integration.

To analyze l-methionine production in shake flask fermentation, one loop of cells was inoculated into a 50 mL tube with 5 mL of LB medium and incubated for 8 h at 37 °C with shaking at 220 rpm on a rotary shaker. Then, 1% (v/v) seed culture was inoculated into 500 mL flasks containing 25 mL of shake flask fermentation medium with 20 g/L glucose, 8 g/L (NH_4_)_2_SO_4_, 2 g/L KH_2_PO_4_, 2 g/L MgSO_4_·7H_2_O, 2.5 g/L yeast extract, 0.02 mg/L VB_12_, 40 g/L MOPS, and 5 mL/L trace element solution, with the pH brought to 7.0 with ammonium solution. Fermentation samples were taken (500 μL each time) for analysis of the substrate and product concentrations.

For fed-batch fermentation in the 5 L bioreactor, one loop of cells was inoculated into a 50 mL tube with 5 mL of LB medium and incubated overnight at 37 °C with shaking at 220 rpm on a rotary shaker. Then, 1% (v/v) of seed culture was inoculated into 500 mL shake flasks containing 50 mL of LB medium and incubated for 8 h at 37 °C with shaking at 220 rpm on a rotary shaker. Then, 100 mL of seed medium was inoculated into a 5 L bioreactor with a final volume of 2 L. Fed-batch fermentation medium was prepared as follows [47]: glucose 10 g/L, (NH_4_)_2_SO_4_ 8 g/L, KH_2_PO_4_ 3 g/L, MgSO_4_·7H_2_O 1 g/L, yeast extract 2 g/L, refined corn pulp (powder) 1 g/L, VB_12_ 0.01 g/L, phosphopyridoxal (PLP) 2 mg/L, and trace element solution 5 mL/L. The pH was maintained at 6.8–7.0 by the addition of 28% (v/v) ammonium. The dissolved oxygen (DO) level was maintained at ~ 30% with agitation at 350 to 800 rpm. When the initial glucose concentration was nearly depleted, the feeding glucose (the stock solution contained 450 g/L glucose, 6 g/L MgSO_4_⋅7H_2_O, 3 g/L KH_2_PO_4_, 33 g/L (NH_4_)_2_SO_4_, and 33.5 g/L ammonium thiosulfate or sodium thiosulfate) was added at an initial rate of 4 g/L/h, which was then gradually increased by 2 g/L/h every 4 h and, finally, maintained at 8 g/L/h until the fermentation process was completed. The glucose concentration in the broth was approximately 0–1 g/L.

The trace element solution was prepared as follows [47]: FeSO_4_·7H_2_O 6 g/L, CaCl_2_ 1.35 g/L, ZnSO_4_·7H_2_O 0.8 g/L, MnSO_4_·4H_2_O 1.5 g/L, CuSO_4_·5H_2_O 0.15 g/L, (NH_4_)_6_Mo_7_O_24_·4H_2_O 0.2 g/L, H_3_BO_3_ 0.1 g/L, CoCl_2_·6H_2_O 0.25 g/L, 35% HCl 10 mL.

### Construction of strains and plasmids

All pTargetF plasmids with different N20 sgRNA sequences were constructed by the Q5 Site-Directed Mutagenesis Kit. All donor DNA templates were obtained by overlap PCR with Q5 Hot Start high-fidelity DNA polymerase. All *E*. *coli* gene knockout, integration, and modulation procedures were performed by the CRISPR/Cas9 genome editing process as reported previously [48]. Transformants were identified by colony PCR, and DNA sequencing was performed at TIANYI HUI YUAN (Beijing).

### Analytical methods

The biomass concentration was monitored by measuring the optical density at 600 nm (OD_600_). Dry cell weight (DCW) was calculated based on the OD_600_ (1 OD_600_ = 0.42 g DCW/L) [49].

Fermentation samples were centrifuged at 8000 × g for 5 min, and the supernatants were used for the analysis of the substrate and product concentrations. The concentration of glucose was assayed with an enzyme electrode analyzer (SBA-40D; Institute of Biology, Shandong, China) containing glucose oxidase [47].

The product concentrations in the supernatant of the fermentation culture were determined by the HPLC method as described previously [50] with slight modifications. An AdvanceBio AAA column (4.6 mm × 150 mm, 5 μm; Agilent Technology, USA) was used at 40 °C after automated online derivatization using *O*-phthalaldehyde (OPA). The elution was performed using a gradient of reagent A (10 mM Na_2_HPO_4_ and 10 mM Na_2_B_4_O_7_, pH to 8.2 by HCl) and reagent B (methanol/acetonitrile/water = 45:45:10, by vol.) at 1 mL/min. The elution gradients were as follows: 0 to 0.35 min, 2% B; then to 13.4 min, 57% B; to 13.5 min, 100% B; 15.7 min, 100% B; 15.8 min, 2% B; 18 min, 2% B. The eluate was monitored at 338 nm.

### SDS-PAGE analysis

The crude proteins from *E. coli* cells were extracted by ultrasonication using lysis buffer (50 mM Tris–HCl (pH 7.5), 1 mM EDTA, 5% glycerol, 1 mM DTT). The supernatants were collected by centrifugation, reconstituted in a loading buffer (1:5 ratio), and heated at 95 °C for 5 min, followed by SDS–polyacrylamide gel electrophoresis. The gel was stained with Coomassie Blue R-250 (Ge Healthcare, Chicago, IL, USA) and scanned using an EPSON Expression 11000XL at a resolution of 300 dpi.

### Constraint-Based metabolic flux analysis

#### Constraint-based metabolic flux analysis

The genome-scale metabolic model *i*Y75_1357 of *E. coli* W3110 was used for the in silico flux metasynthetic reaction. A synthetic reaction route for 2-ketobutyrate by the *metB*-encoding cystathionine γ-synthase was added to *i*Y75_1357 to synthesize 2-ketobutyrate from *O*-succinyl-l-homoserine. The following reaction was added to iY75_1357: ‘suchms_c + h2o_c → 2obut_c + succ_c + nh4_c + h_c’. In silico analysis of the modified target for overproduction of l-methionine was performed with MATLAB 2014a (The MathWorks, Natick, MA, USA) and the COBRA Toolbox 2.05 (MathWorks Inc. PortolaValley, CA, USA) with the *glpk* solver [51]. Constraint-based flux balance analysis was carried out by the linear programming-based optimization of growth or l-methionine biosynthesis flux as an objective cellular function. The glucose uptake rate was set to 100 mmol/gCDW/h. Small-molecular metabolites such as CO_2_, H_2_O, SO_3_, NH_3_, PO_4_, and O_2_ were allowed to be freely transported across the cell membrane.

#### RNA preparation and quantitative real-time RT-PCR

*E. coli* strains were cultivated in LB medium as described above. After overnight culture, cells were harvested by centrifugation. Total RNA was isolated using the RNA prep Pure Cell/Bacteria Kit (Tiangen, China). Reverse transcription of approximately 300 ng of RNA was performed using the FastQuant RT Kit (Tiangen, China) with the specific primers listed in Additional file 1: Table S2. GoTaq qPCR Master Mix (Promega, Madison, WI, USA) in a 20 µL volume was used to perform quantitative PCR using the LightCycler® 96 Real-Time PCR System (Roche, Basel, Switzerland). The GAPDH gene was used as the reference gene to normalize the mRNA levels of *cysD*, *cysN*, *cysH*, *cysC*, *cysJ*, *cysI*, and *cysP*. Negative controls in each PCR were run to exclude DNA and other contaminants. The qPCR products were verified by melting curve analysis. Data were analyzed using LightCycler^®^ 96 software (Roche, Basel, Switzerland) according to the 2^−∆∆CT^ method [52].

## Supplementary Information

**Additional file 1: Table S1.** Plasmids used in this study. **Table S2.** Primers used in this study. **Figure S1.** The expression of wild-type and mutated MetA was detected by SDS-PAGE in MET1. Lane 0, MET1/pBR322. Lane 1, MET1/pBR322-*metA*. Lane 2, MET1/pBR322-*metA*^I124L^. Lane 3, MET1/pBR322-*metA*^I229Y^. Lane 4, MET1/pBR322-*metA*^I124L, I229Y^_._ Lane 5, MET1/pBR322-*metA*^R27C, I296S, P298L^. Lane 6, MET1/pBR322-*metA*^R27C, I296S, P298L, I124L^. Lane 7, MET1/pBR322-*metA*^R27C, I296S, P298L, I229Y^. Lane 8, MET1/pBR322-*metA*^R27C, I296S, P298L, I124L, I229Y^. **Figure S2.** l-methionine relative titer and OD_600_ of MET4, MET5, MET6, and MET7. Data are presented by mean values with the standard deviation from three biological replicates. One-way analysis of variance was used to determine significant differences. **Figure S3.** Fed-batch fermentation process curve in a 5 L bioreactor. With ammonium thiosulfate feeding during the fed-batch cultivation of the MET17 strain. Data are presented as the mean values with the standard deviation from two replicates.

## Data Availability

All data generated or analyzed during this study are included in this published article (and its additional files).

## Abbreviations

CRISPR: Clustered Regularly Interspaced Short Palindromic Repeat
*E. coli*: *Escherichia coli*
FBA: Flux balance analysis
HPLC: High-performance liquid chromatography
WT: Wild type
ATP: Adenosine triphosphate
NADPH: Reduced form of nicotinamide adenine dinucleotide phosphate
OD_600_: Optical density at wavelength (λ) 600 nm
