# Supplementary material for: Multivariate modular metabolic engineering for enhanced l-methionine biosynthesis in Escherichia coli
Source: Biotechnol Biofuels Bioprod. 2023 Jun 13;16:101. doi: 10.1186/s13068-023-02347-7 (PMC10265765; doi:10.1186/s13068-023-02347-7)
Supplement: Supplementary file 1 — Additional file 1: Table S1. Plasmids used in this study. Table S2. Primers used in this study. Figure S1. The expression of wild-type and mutated MetA was detected by SDS-PAGE in MET1. Lane 0, MET1/pBR322. Lane 1, MET1/pBR322-metA. Lane 2, MET1/pBR322-metAI124L. Lane 3, MET1/pBR322-metAI229Y. Lane 4, MET1/pBR322-metAI124L, I229Y. Lane 5, MET1/pBR322-metAR27C, I296S, P298L. Lane 6, MET1/pBR322-metAR27C, I296S, P298L, I124L. Lane 7, MET1/pBR322-metAR27C, I296S, P298L, I229Y. Lane 8, MET1/pBR322-metAR27C, I296S, P298L, I124L, I229Y. Figure S2. l-methionine relative titer and OD600 of MET4, MET5, MET6, and MET7. Data are presented by mean values with the standard deviation from three biological replicates. One-way analysis of variance was used to determine significant differences. Figure S3. Fed-batch fermentation process curve in a 5 L bioreactor. With ammonium thiosulfate feeding during the fed-batch cultivation of the MET17 strain. Data are presented as the mean values with the standard deviation from two replicates. [file 13068_2023_2347_MOESM1_ESM.docx]

**Supplementary Information**

For

**Multivariate modular metabolic engineering for enhanced l-methionine biosynthesis in *Escherichia coli***

**Zhongcai Li^1,2^** **^†^, Qian Liu^1,3^** **^†^, Jiahui Sun^1,4^, Jianjian Sun^1^, Mingjie Li^1,2^, Yun Zhang^1^, Aihua Deng^1^, Shuwen Liu^1*^ and Tingyi Wen^1,5,6*^**

1. State Key Laboratory of Microbial Resources, Institute of Microbiology, Chinese Academy of Sciences, Beijing 100101, China

2. University of Chinese Academy of Sciences, Beijing 100049, China

3. National Laboratory of Biomacromolecules, Institute of Biophysics, Chinese Academy of Sciences, Beijing 100101, China.

4. College of Life Sciences, Hebei University, Baoding, 071002, China

5. Savaid Medical School, University of Chinese Academy of Sciences, Beijing 100049, China

6. China Innovation Academy for Green Manufacture, Chinese Academy of Sciences, Beijing 100049, China

* Correspondence: liusw@im.ac.cn, [wenty@im.ac.cn](mailto:wenty@im.ac.cn)

**^†^** Zhongcai Li and Qian Liu contributed equally to this work

**Table S1** **Plasmids used in this study**.

| **Plasmids** | **Relevant characteristics** | **sources** |
| --- | --- | --- |
| pCas9 | *repA101(Ts )kan Pcas-cas 9ParaB-Red lacIq*  *Ptrc-sgRNA-pMB1* | [1] |
| pTargetF | vector backbone for expressing sgRNA, *Pmb1ori* Amp^R,^ | [1] |
| pBR322 | Amp^R^, Tet^R^ | New England Biolabs |
| pACYC184 | Tet^R^, Cm^R^ | New England Biolabs |
| p1 | pBR322-metA | This study |
| p2 | pBR322-*metA*^I124L^ | This study |
| p3 | pBR322-*metA*^I229Y^ | This study |
| p4 | pBR322-*metA*^I124L, I229Y^ | This study |
| p5 | pBR322-*metA*^R27C, I296S, P298L^ | This study |
| p6 | pBR322-*metA*^R27C, I124L, I296S, P298L^ | This study |
| p7 | pBR322-*metA*^R27C, I229Y, I296S, P298L^ | This study |
| p8 | pBR322-*metA*^R27C, I124L, I229Y, I296S, P298L^ | This study |
| p9 | pACYC184-*cysD* | This study |
| p10 | pACYC184-*cysN* | This study |
| p11 | pACYC184-*cysDN* | This study |
| p12 | pACYC184-*cysC* | This study |
| p13 | pACYC184-*cysH* | This study |
| p14 | pACYC184-*cysJ* | This study |
| p15 | pACYC184-*cysI* | This study |
| p16 | pACYC184-*cysJI* | This study |
| pTargetF-*metJ* | pTargetF carrying sgRNA with an N20 sequence for targeting the *metJ* locus, N20+PAM:  CTGACGACGCGTGCGTTCATCGG | This study |
| pTargetF-*metA* | pTargetF carrying sgRNA with an N20 sequence for targeting the *metA* locus, N20+PAM:  GCCGCGCTCAATATCCTCTACGG | This study |
| pTargetF-*pykA* | pTargetF carrying sgRNA with an N20 sequence for targeting the *pykA* locus, N20+PAM:  CAAACTTGGCGGCGGTTTGTCGG | This study |
| pTargetF-*pykF* | pTargetF carrying sgRNA with an N20 sequence for targeting the *pykF* locus, N20+PAM:  GAGCACCTGAAAGCGCACGGCGG | This study |
| pTargetF-MetD | pTargetF carrying sgRNA with an N20 sequence for targeting the MetD locus, N20+PAM:  AATTGAAGCTTCCCGCGCAATGG | This study |
| pTargetF-P*_metBL_*_::_P*_trc_* | pTargetF carrying sgRNA with an N20 sequence for targeting the *metBL* locus, N20+PAM:  CATTTCACCGACAAAGCCCAGGG | This study |
| pTargetF-P*_metC_*_::_P*_trc_* | pTargetF carrying sgRNA with an N20 sequence for targeting the *metC* locus, N20+PAM:  GCGGGCGCATCCTGAAGAATTGG | This study |
| pTargetF-P*_metF_*_::_P*_trc_* | pTargetF carrying sgRNA with an N20 sequence for targeting the *metF* locus, N20+PAM:  TTTACATCTGGACGTCTAAACGG | This study |
| pTargetF-*rhtA*::*metA*^fbr^ | pTargetF carrying sgRNA with an N20 sequence for targeting the *rhtA*::*metA*^fbr^ locus, N20+PAM:  AGTCATATTGCTCGTTGCCATGG | This study |
| pTargetF-*rhtA*::*metC* | pTargetF carrying sgRNA with an N20 sequence for targeting the *rhtA*::*metC* locus, N20+PAM:  CGATTTAACCGGCTGTGCGCTGG | This study |
| pTargetF-*rhtA*::*cysE*^fbr^ | pTargetF carrying sgRNA with an N20 sequence for targeting the *rhtA*::*cysE*^fbr^ locus, N20+PAM:  CGCTATCATCGCCGCTTCAATGG | This study |
| pTargetF-*rhtA*::*serA*^fbr^ | pTargetF carrying sgRNA with an N20 sequence for targeting the *rhtA*::*serA*^fbr^ locus, N20+PAM:  CGGTTACCGCTGTTGTTTTACGG | This study |
| pTargetF-*ygaY*::*cysDN* | pTargetF carrying sgRNA with an N20 sequence for targeting the *ygaY*::*cysDN* locus, N20+PAM:  CCTCAACTACCCACAGTTGTTGG | This study |
| pTargetF-*sdaA* | pTargetF carrying sgRNA with an N20 sequence for targeting the *sdaA* locus, N20+PAM:  ATGCAGGCATGTATCGATCGCGG | This study |
| pTargetF-*yhaM* | pTargetF carrying sgRNA with an N20 sequence for targeting the *yhaM* locus, N20+PAM:  TTATCGCAGGAAGGTTTGAGCGG | This study |

*kan* kanamycin resistance gene, Amp ampicillin, Tet tetracycline, Cm chloramphenicol, R resistance.

**Table S2 Primers used in this study**

| **Primer** | **Sequences（5’-3’）** | **Description** |
| --- | --- | --- |
| pTargetF-*metJ*-F | GACGAAATCCGTTTTAGAGCTAGAAATAGC | Construction of pTargetF-*metJ* |
| pTargetF-*metJ*-R | GCTGCGCTCTGACTAGTATTATACCTAGGACTGAGC |  |
| pTargetF-*metA*-F | ATATCCTCTAGTTTTAGAGCTAGAAATAGC | Construction of pTargetF-*metA* |
| pTargetF-*metA*-R | TGAGCGCGGCGACTAGTATTATACCTAGGACTGAGC |  |
| pTargetF-*pykF*-F | AAGCGCACGGGTTTTAGAGCTAGAAATAGC | Construction of pTargetF-*pykF* |
| pTargetF-*pykF*-R | TCAGGTGCTCGACTAGTATTATACCTAGGACTGAGC |  |
| pTargetF-*pykA*-F | GGCGGTTTGTGTTTTAGAGCTAGAAATAGC | Construction of pTargetF-*pykA* |
| pTargetF-*pykA*-R | GCCAAGTTTGGACTAGTATTATACCTAGGACTGAGC |  |
| pTargetF-MetD-F | TCCCGCGCAAGTTTTAGAGCTAGAAATAGC | Construction of pTargetF-MetD |
| pTargetF-MetD-R | AGCTTCAATTGACTAGTATTATACCTAGGACTGAGC |  |
| pTargetF-*metBL*-F | ACAAAGCCCAGTTTTAGAGCTAGAAATAGC | Construction of ptargetF-P*_metBL_*::P*_trc_* |
| pTargetF-*metBL*-R | CGGTGAAATGGACTAGTATTATACCTAGG |  |
| pTargetF-*metC*-F | CCTGAAGAATGTTTTAGAGCTAGAAATAGC | Construction of pTargetF-P*_metC_*::P*_trc_* |
| pTargetF-*metC*-R | ATGCGCCCGCGACTAGTATTATACCTAGG |  |
| pTargetF-*metF*-F | GACGTCTAAAGTTTTAGAGCTAGAAATAGC | Construction of ptargetF-P*_metF_*::P*_trc_* |
| pTargetF-*metF*-R | CAGATGTAAAGACTAGTATTATACCTAGG |  |
| pTargetF-*rhtA*1-F | TCGTTGCCAGTTTTAGAGCTAGAAATAGC | Construction of pTargetF-*rhtA*::*metA*^fbr^ |
| pTargetF-*rhtA*1-R | GCAATATGACTGACTAGTATTATACCTAGG |  |
| pTargetF-*rhtA*2-F | GCTGTGCGCGTTTTAGAGCTAGAAATAGC | Construction of pTargetF-*rhtA*::*metC* |
| pTargetF-*rhtA*2-R | CGGTTAAATCGGACTAGTATTATACCTAGG |  |
| pTargetF-*rhtA*3-F | GCCGCTTCAAGTTTTAGAGCTAGAAATAGC | Construction of pTargetF-*rhtA*:: P*_23119_*-*cysE*^fbr^ |
| pTargetF-*rhtA*3-R | GATGATAGCGGACTAGTATTATACCTAGGACTGAGC |  |
| pTargetF-*rhtA*4-F | TGTTGTTTTAGTTTTAGAGCTAGAAATAGC | Construction of pTargetF-*rhtA*::P*_23105_*-*serA*^fbr^ |
| pTargetF-*rhtA*4-R | GCGGTAACCGGACTAGTATTATACCTAGGACTGAGC |  |
| pTargetF-*ygaY*-F | CCACAGTTGTGTTTTAGAGCTAGAAATAGC | Construction of pTargetF-*ygaY*::P*_trc_*-*cysDN* |
| pTargetF-*ygaY*-R | GTAGTTGAGGGACTAGTATTATACCTAGGACTGAGC |  |
| pTargetF-*sdaA*-F | GTATCGATCGGTTTTAGAGCTAGAAATAGC | Construction of pTargetF-*sdaA* |
| pTargetF-*sdaA*-R | ATGCCTGCATGACTAGTATTATACCTAGGACTGAGC |  |
| pTargetF-*yhaM*-F | AAGGTTTGAGGTTTTAGAGCTAGAAATAGC | Construction of pTargetF-*yhaM* |
| pTargetF-*yhaM*-R | CCTGCGATAAGACTAGTATTATACCTAGGACTGAGC |  |
| *metJ*-F | CTGTTCGTCGTCATTTAACCCGCTAC | Upstream editing template of *metJ* knockout |
| *metJ*-R | ACTCCGCGCCGCTCTTTTTTGCTGAGATACTTAATCCTCTTCGTCA |  |
| *metJ*-F1 | TGACGAAGAGGATTAAGTATCTCAGCAAAAAAGAGCGGCGCGGAGT | Downstream editing template of *metJ* knockout |
| *metJ*-R1 | GTGGCTGCCAGGCAAAGGCAGGTAC |  |
| *metJ*-F2 | CGCGCGGTTCATTAAATCCGG | *metJ* identification |
| *metJ*-R2 | GCGTATCACCAGCATACCGGTG |  |
| *metA*-F | CCAAGCTTTTTCTCTCCTTTTAGTCATTC (*Hind III*) | Amplification of *metA* |
| *metA*-R | CGGAATTCCAGCTCCTCGTCATGG (EcoR I) |  |
| *metA*-F1 | ACTCCAGCACCTGTTTGAGCTGCGGCCAGTAAGCGAC | I124L |
| *metA*-R1 | GTCGCTTACTGGCCGCAGCTCAAACAGGTGCTGGAGT |  |
| *metA*-F2 | GATGGCCCGTCACAAAGGCATAGCGCTTATCTTTACTGGC | I229Y |
| *metA*-R2 | GCCAGTAAAGATAAGCGCTATGCCTTTGTGACGGGCCATC |  |
| *metA*-F3 | GTGGACGAATTTCCTGACCAGACGCACAAGAAGTTGTCATCACAAAGACG | R27C |
| *metA*-R3 | CGTCTTTGTGATGACAACTTCTTGTGCGTCTGGTCAGGAAATTCGTCCAC |  |
| *metA*-F4 | GGATTCATGTGCCGTAGATCGTATAGCGTGCTCTGGTAGACGTAATAGTTG | I296S  P298L |
| *metA*-R4 | CAACTATTACGTCTACCAGAGCACGCTATACGATCTACGGCACATGAATCC |  |
| *metA*-F5 | CAACCGCCTGCTCATTTTG | Downstream editing template of *metA*^fbr^ |
| *metA*-R5 | TATCTCTACGCGGCGGTCTT |  |
| *metA*-F6 | CTGACGACGCGTGCGTTCATGATCTACGGCACATGAATCCAACGC | Amplification of template for *metA* knockout |
| *metA*-R6 | ATGAACGCACGCGTCGTCAGGTTGTCATCACAAAGACGTTTTCTT |  |
| *metA*-F7 | CACCTCAGAGCGTCCCGTAA | *metA*^fbr^ identification |
| *metA*-R7 | GTTTATTGCGTTGTGGCGTA |  |
| pBR322-F | AAGTGCCACCTGACGTCTAA | pBR322 identification |
| pBR322-R | GCCTGCCACCATACCCAC |  |
| *pykA*-F | CGCCATCGCGGCGTTATTTCATTCGGATTTCATGTTCAAGCAACACCTGGTTGTTTCAGTCAACGGAGTATTACGATGAGTACCGTGGG | Amplification of template for *pykA* knockout |
| *pykA*-R | TGGCGTTTTCGCCGCATCCGGCAACGTACTTACTCTACCGTTAAAATACGCGTGGTATTAGTAGAACCCACGGTACTCATCGTAATACTC |  |
| *pykF*-F | CTTTCGTAATACCGGATTCGCTTTCCGGCAGTGCGCCCAGAAAGCAAGTTTCTCCCATCCTTCTCAACTTAAAGACTAAGACTGTCTATTGCTTTTG | Amplification of template for *pykF* knockout |
| *pykF*-R | GATTTTTTATTGCTTCTGGTTATCGATTAAATAAAAAAAGCGCCCATCAGGGCGCTTCGATATACAAATTAATTCACAAAAGCAATAGACAGTCTTAG |  |
| *pykA*-F1 | GCTGCAACGCATGAGTTGTATG | *pykA* identification |
| *pykA*-R1 | CATTCATCCAGTCGGTACGTCAG |  |
| *pykF*-F1 | CCAGTGCGTTAATGACGGTTTCC | *pykF* identification |
| *pykF*-R1 | GCGGTATTTAGTAGCCATGTTGTC |  |
| MetD-F | GGCAGGTCTGCCAGGCTATT | Amplification of template for MetD knockout |
| MetD-R | CATGGAACAGCCGCTTAGCAATCCAGACGTCTAAATCAAT |  |
| MetD-F1 | ATTGATTTAGACGTCTGGATTGCTAAGCGGCTGTTCCATG |  |
| MetD-R1 | AGGCGGCTGGCGTCCGACTG |  |
| *yjeH*-F | GCGAAACTCTTCAACACTAC | Upstream editing template of *yjeH* |
| *yjeH*-R | TTTCTTAGACGTCGGAATTGAGTTAGTCGTGGCATCTCG |  |
| P*_L_*-F | CGAGATGCCACGACTAACTCAATTCCGACGTCTAAGAAA | Amplification of P*_L_* promoter |
| P*_L_*-R | ATGGGTTGATGTCCGATTGCGGTCAGTGCGTCCTGCTGAT |  |
| *yjeH*-F1 | ATCAGCAGGACGCACTGACCGCAATCGGACATCAACCCAT | Amplification of *yjeH* |
| *yjeH*-R1 | TGCTTCGTAAACTTCGTCAGATGTCACTGGTACGTAACCA |  |
| *yjeH*-F2 | TGGTTACGTACCAGTGACATCTGACGAAGTTTACGAAGCA | Downstream editing template of *yjeH* |
| *yjeH*-R2 | TGACTTCGCAGCTATGCAGT |  |
| MetD-F2 | GTACGCACCAGCACTTTTGT | MetD and P*_L_*-*yjeH* operons identification |
| MetD-R2 | TGCTCGAACTGGAAACTGCT |  |
| *metBL*-F | ATGTATTGACGTCCATTAACACAATGTTTACTCTGGTGCCTGACATTTCACCGACAAAG | Amplification of template for P*_metBL_*::P*_trc_* |
| *metBL*-R | TCCACACATTATACGAGCCGGATGATTAATTGTCAACTTTGTCGGTGAAATGTC |  |
| *metBL*-F1 | GCTCGTATAATGTGTGGACAGGGAACTTCATCACATGACGCGTAAACA |  |
| *metBL*-R1 | TTCGTCGTCATTTAACCCGCTACGCACTGCGATGGTGGCCTGTTTACGCGTCATGTGAT |  |
| *metBL*-F2 | GCTATTTGGGATTTGCTCAATC | P*_metBL_*::P*_trc_* identification |
| *metBL*-R2 | GTTATAGGTGCTGGAAAGATG |  |
| P*_metC_*-F | CGTTTACGCAGTAAAAAAGTCACCAGCACGCCATTTGCGAAAATTTTCTGCTTTATGCC | Amplification of template for P*_metC_*::P*_trc_* |
| P*_metC_*-R | TCCACACATTATACGAGCCGGATGATTAATTGTCAAGGCATAAAGCAGAAAATTTTCG |  |
| P*_metC_*-F1 | GCTCGTATAATGTGTGGAGCGAATATTCATGCTAGTTTAGACATCCAG |  |
| P*_metC_*-R1 | GCTTTTTGTCCGCCATGTCGGGATTCCTGTTTTTATACGTCTGGATGTCTAAACTAGCA |  |
| P*_metC_*-F2 | CGACGTCGAATTGATAGTC | P*_metC_*::P*_trc_* identification |
| P*_metC_*-R2 | CCGAGAGTGTATTTTTTGCTG |  |
| *metF*-F | TTGGTCATTTTTCGGTTGACGCCCTTCGGCTTTTCCTTCATCTTTACATCTGGACGTCT | Amplification of template for P*_metF_*::P*_trc_* |
| *metF*-R | TCCACACATTATACGAGCCGGATGATTAATTGTCAAAGACGTCCAGATGTAAAG |  |
| *metF*-F1 | GCTCGTATAATGTGTGGAGATAGATGTGCACAACACAACATATAACTACAAG |  |
| *metF*-R1 | GGCGTGAAAAAAGCTCATACCTTACCTCATCAATCGCTTGTAGTTATATGTTGTGTTG |  |
| *metF*-F2 | GTCCGGTTTGCTTTGCATAC | P*_metF_*::P*_trc_* identification |
| *metF*-R2 | GTTTCGTTCGAGTTTTTCCC |  |
| *metA*-O-F | CATAACCACCTCAAATGTGATTC | Upstream editing template of *metA*^fbr^ operon |
| *metA*-O-R | TCCACACATTATACGAGCCGGATGATTAATTGTCAACTAGACTTAAGTAAAGCGTGGA |  |
| *metA*-O-F1 | TTGACAATTAATCATCCGGCTCGTATAATGTGTGGATGTAGTGAGGTAATCAGGT | Amplification of *metA*^fbr^ operon |
| *metA*-O-R1 | CTTAGCTAACGAGGCTCCACCTGCTGAGGTACGTTTCGGA |  |
| *metA*-O-F2 | TCCGAAACGTACCTCAGCAGGTGGAGCCTCGTTAGCTAAG | Downstream editing template of *metA*^fbr^ operon |
| *metA*-O-R2 | GATAACAGGGTAATAGATCTACAGGAACCACAGACCAAGAAC |  |
| *metA*-O-F3 | CGGTGAAAGCCAGAACTG | *metA*^fbr^ operon identification |
| *metA*-O-R3 | CAATTGGTTCGTTGATTGCAG |  |
| *metC*-F | GTGGAGCCTCGTTAGCTAAG | Upstream editing template of *metC* operon |
| *metC*-R | TCCACACATTATACGAGCCGGATGATTAATTGTCAACAGGAACCACAGACCAAGAAC |  |
| *metC*-F1 | TTGACAATTAATCATCCGGCTCGTATAATGTGTGGAGTTTAGACATCCAGACGT | Amplification of *metC* operon |
| *metC*-R1 | CTGCAATCAACGAACCAATTGGACTTTTCACAATAAAATGTCTGC |  |
| *metC*-F2 | GCAGACATTTTATTGTGAAAAGTCCAATTGGTTCGTTGATTGCAG | Downstream editing template of *metC* operon |
| *metC*-R2 | GATAACAGGGTAATAGATCTACTCTCTTTGCGTACTGTCAG |  |
| *metC-*F3 | GTGGAGCCTCGTTAGCTAAG | *metC* operon identification |
| *metC*-R3 | CATGGTTATGCATAACCATGCA |  |
| *cysE*-F | GCAGACATTTTATTGTGAAAAGTCCAATTGGTTCGTTGATTGCAG | Upstream editing template of *cysE*^fbr^ operon |
| *cysE*-R | GCTAGCATTATACCTAGGACTGAGCTAGCTGTCAATGTCAGTGTTTCTCCGAGG |  |
| *cysE*-F1 | TTGACAGCTAGCTCAGTCCTAGGTATAATGCTAGCCTCATCGTGTGGAGTAAGCA | For amplification of *cysE*^fbr^ operon |
| *cysE*-R1 | ATTCGTGAAGGTGTGCGTATTGGCGCG |  |
| *cysE*-F2 | CGCGCCAATACGCACACCTTCACGAAT |  |
| *cysE*-R2 | CTCTCTTTGCGTACTGTCAGGAGCGGTATTGAGAGATTAG |  |
| *cysE*-F3 | CTAATCTCTCAATACCGCTCCTGACAGTACGCAAAGAGAG | Downstream editing template of *cysE*^fbr^ operon |
| *cysE*-R3 | CTGGCTGTTCCTATCACACTAATAG |  |
| *cysE*-F4 | GGACCTTGATTCGCCTGCATATTG | *cysE*^fbr^ operon identification |
| *cysE*-R4 | GGATAACCTGGCGATTCAGCAAC |  |
| *serA*-F | TCCGAAACGTACCTCAGCAGGTGGAGCCTCGTTAGCTAAG | Upstream editing template of *serA*^fbr^ operon |
| *serA*-R | GCTAGCATAGTACCTAGGACTGAGCTAGCCGTAAACCGTTGCTCTTTGGCAAAGC |  |
| *serA*-F1 | TTTACGGCTAGCTCAGTCCTAGGTACTATGCTAGCCAAATCCGCACACAACATTTC | Amplification of *serA*^fbr^ operon |
| *serA*-R1 | GTTCAGCGCAGTTAGCACGCCCGGACGAGCTTCAGCGATGTGCATCAGACGAC |  |
| *serA*-F2 | GCTCGTCCGGGCGTGCTAACTGCGCTGAACAAAATCTTC |  |
| *serA*-R2 | TCCCACCCAGCGAAACGGTGAGGGCTTTACCGTTAC |  |
| *serA*-F3 | GTAACGGTAAAGCCCTCACCGTTTCGCTGGGTGGGA | Downstream editing template of *serA*^fbr^ operon |
| *serA*-R3 | CAGGAACCACAGACCAAGAAC |  |
| *serA*-F4 | GCCATACGATCTACGGCACA | *serA*^fbr^ operon identification |
| *serA*-R4 | CTATTTACCGCGCCGAGAGT |  |
| 184-*cysD*-F | AATCAGATAAAATATTTCTAGATTGACAATTAATCATCCGGCTCGTATAATGTGTGGATGCAAAGGAACGGTTATG | Construction of p9-p16 plasmids |
| 184-*cysD*-R | CACGATGCGTCCGGCGTAGAGGATCCAGTGCGGTGTTCATCTTAAAAA |  |
| 184-*cysN*-F | AATCAGATAAAATATTTCTAGATTGACAATTAATCATCCGGCTCGTATAATGTGTGGAGTCAGGGGTATTTTTAAGATG |  |
| 184-*cysN*-R | CACGATGCGTCCGGCGTAGAGGATCCGCAGCGCCATTATTTATC |  |
| 184-*cysC*-F | AATCAGATAAAATATTTCTAGATTGACAATTAATCATCCGGCTCGTATAATGTGTGGATTTGCTGGGGGATAAATAATG |  |
| 184-*cysC*-R | CACGATGCGTCCGGCGTAGAGGATCCAAACCCGGTGGTGTCTCAGG |  |
| 184-*cysH*-F | AATCAGATAAAATATTTCTAGATTGACAATTAATCATCCGGCTCGTATAATGTGTGGAGCAAACAGTGAGGAATCTATG |  |
| 184-*cysH*-R | CACGATGCGTCCGGCGTAGAGGATCCTAATTTGTCCGGCAATATTTACC |  |
| 184-*cysJ*-F | TCAGATAAAATATTTCTAGATTGACAATTAATCATCCGGCTCGTATAATGTGTGGAACTGGAACATAACGACGCATGAC |  |
| 184-*cysJ*-R | CACGATGCGTCCGGCGTAGAGGATCCCTGGATGTTTTTCGCTCATTAGTAG |  |
| 184-*cysI*-F | AATCAGATAAAATATTTCTAGATTGACAATTAATCATCCGGCTCGTATAATGTGTGGACAGCGAGATGTCTACTA |  |
| 184-*cysI*-R | CACGATGCGTCCGGCGTAGAGGATCCACAAGACCGGGCTGATGGTTAATCC |  |
| *cysDN*-F | CCTACAAACCACATCGCACATTTC | Upstream editing template of *cysDN* operon |
| *cysDN*-R | CATTATACGAGCCGGATGATTAATTGTCAAAAGCAACCCAAAAGACGGTGCGCCAGCC |  |
| *cysDN*-F1 | AATCATCCGGCTCGTATAATGTGTGGATGCAAAGGAACGGTTATGGATCAAATACG | Amplification of *cysDN* |
| *cysDN*-R1 | GCAGCGCCATTATTTATCCCCCAGCAAATCG |  |
| *cysDN*-F2 | CTGGGGGATAAATAATGGCGCTGCCTTGCCGCTCCACCTTTTAACTACAGCGATG | Downstream editing template of *cysDN* operon |
| *cysDN*-R2 | GGAGTAGGGCTTTCCATAGAGTGTAC |  |
| *cysDN*-F3 | GTTTTTGCCGCATCCGCCGTTCTG | *cysDN* operon identification |
| *cysDN*-R3 | GCATTCAGACCGAACGCAAAGGCC |  |
| pTargetF-F | GCCTTTTGCTCACATGTTCT | pTargetF identification |
| pTargetF-F | GTAGGGATAACAGGGTAATAGA |  |
| pCas9-F | ATTAATGAATCGGCCAACGC | pCas9 identification |
| pCas9-R | CCAGTAAAGCGGCGGTGCACA |  |
| GAPDH-RT-F | TTGATGGCCCGTCTCACAAA | Real-time quantitative PCR |
| GAPDH-RT-R | GACGAACGGTCAGGTCAACT |  |
| *cysC*-RT-F | TCGGTTTTAGCGATGCCGAT |  |
| *cysC*-RT-R | TCGATAAAGCGCCCTTCTCC |  |
| *cysD*-RT-F | TGACAACCGTATCGACCTGC |  |
| *cysD*-RT-R | ATTTCCGGCAGTGTTTGTGC |  |
| *cysH*-RT-F | GCAATTCAGCGTGGCGTATT |  |
| *cysH*-RT-R | ACGGGTTGTATGGGTATCGC |  |
| *cysI*-RT-F | AGCATCTGTTGCCTCGTACC |  |
| *cysI*-RT-R | TCGATATCGTTCTGTGGCGG |  |
| *cysJ*-RT-F | TATCGATCTTGCCTGGTCGC |  |
| *cysJ*-RT-R | CATGCCACCAAATTCGGCAA |  |
| *cysN*-RT-F | TGAGATCGACATCAGCCGTG |  |
| *cysN*-RT-R | AACTCTGCCCTGGAGAAAGC |  |

**
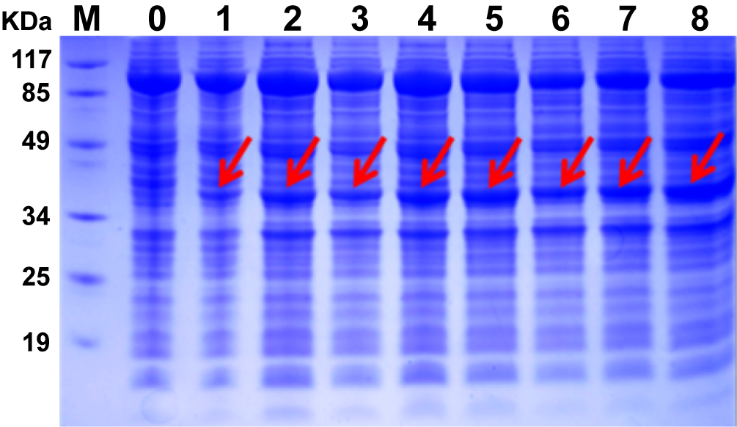
**

**Figure S1**. **The expression of wild-type and mutated MetA was detected by SDS-PAGE in MET1**. Lane 0, MET1/pBR322. Lane 1, MET1/pBR322-*metA*. Lane 2, MET1/pBR322-*metA*^I124L^. Lane 3, MET1/pBR322-*metA*^I229Y^. Lane 4, MET1/pBR322-*metA*^I124L, I229Y^_._ Lane 5, MET1/pBR322-*metA*^R27C, I296S, P298L^. Lane 6, MET1/pBR322-*metA*^R27C, I296S, P298L, I124L^. Lane 7, MET1/pBR322-*metA*^R27C, I296S, P298L, I229Y^. Lane 8, MET1/pBR322-*metA*^R27C, I296S, P298L, I124L, I229Y^.

**
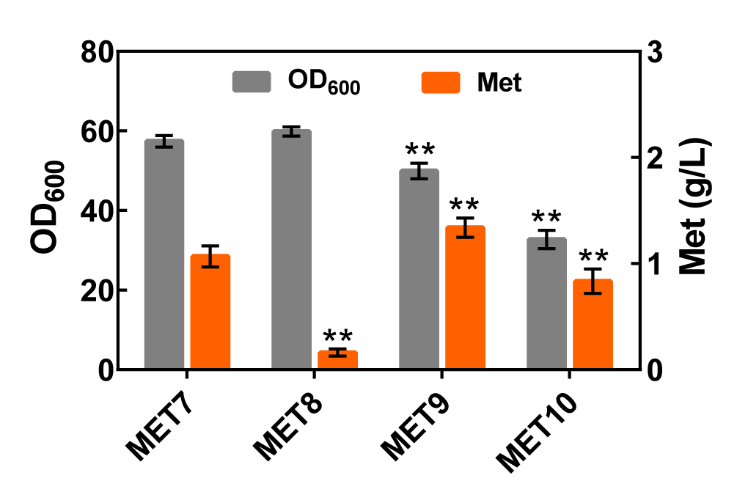
**

**Figure S2**. **l-Methionine relative titer and OD_600_ of MET4, MET5, MET6, and MET7**. Data are presented by mean values with the standard deviation from three biological replicates. One-way analysis of variance (ANOVA) was used to determine significant differences (* *p* < 0.05, ** *p* < 0.01).


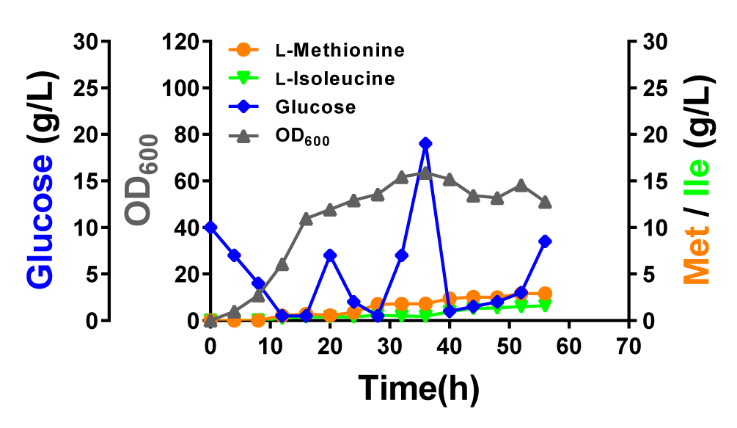


**Figure S3. Fed-batch fermentation process curve in a 5 L bioreactor**. With ammonium thiosulfate (60 g/L) feeding during the fed-batch cultivation of the MET17 strain. Data are presented as the mean values with the standard deviation from two replicates.

**References**

1. Jiang Y, Chen B, Duan C, Sun B, Yang J, Yang S: Multigene editing in the *Escherichia coli* genome via the CRISPR-Cas9 system. Appl Environ Microbiol 2015, 81:2506-2514.
